# New Role for Interleukin‐13 Receptor α1 in Myocardial Homeostasis and Heart Failure

**DOI:** 10.1161/JAHA.116.005108

**Published:** 2017-05-20

**Authors:** Uri Amit, David Kain, Allon Wagner, Avinash Sahu, Yael Nevo‐Caspi, Nir Gonen, Natali Molotski, Tal Konfino, Natalie Landa, Nili Naftali‐Shani, Galia Blum, Emmanuelle Merquiol, Danielle Karo‐Atar, Yariv Kanfi, Gidi Paret, Ariel Munitz, Haim Y. Cohen, Eytan Ruppin, Sridhar Hannenhalli, Jonathan Leor

**Affiliations:** ^1^ Neufeld Cardiac Research Institute Tel Aviv University Tel‐Hashomer Israel; ^2^ Sheba Center for Regenerative Medicine, Stem Cell, and Tissue Engineering Sheba Medical Center Tel‐Hashomer Israel; ^3^ Tamman Cardiovascular Research Institute Sheba Medical Center Tel‐Hashomer Israel; ^4^ The Dr. Pinchas Borenstein Talpiot Medical Leadership Program Sheba Medical Center Tel‐Hashomer Israel; ^5^ The Blavatnik School of Computer Science Tel Aviv University Tel Aviv Israel; ^6^ Department of Clinical Microbiology and Immunology Sackler School of Medicine Tel Aviv University Tel Aviv Israel; ^7^ The Blavatnik School of Computer Science and Sackler School of Medicine Tel Aviv University Tel Aviv Israel; ^8^ Department of Electrical Engineering and Computer Science University of California Berkeley CA; ^9^ Department of Cell Biology and Molecular Genetics Center for Bioinformatics and Computational Biology University of Maryland College Park MD; ^10^ Department of Pediatric Critical Care Medicine Safra Children's Hospital Tel‐Hashomer Israel; ^11^ The Institute of Drug Research The School of Pharmacy The Faculty of Medicine Campus Ein Karem Hebrew University Jerusalem Israel; ^12^ Mina & Everard Goodman Faculty of Life Sciences Bar‐Ilan University Ramat Gan Israel

**Keywords:** cytokine, heart failure, receptor, Growth Factors/Cytokines

## Abstract

**Background:**

The immune system plays a pivotal role in myocardial homeostasis and response to injury. Interleukins‐4 and ‐13 are anti‐inflammatory type‐2 cytokines, signaling via the common interleukin‐13 receptor α1 chain and the type‐2 interleukin‐4 receptor. The role of interleukin‐13 receptor α1 in the heart is unknown.

**Methods and Results:**

We analyzed myocardial samples from human donors (n=136) and patients with end‐stage heart failure (n=177). We found that the interleukin‐13 receptor α1 is present in the myocardium and, together with the complementary type‐2 interleukin‐4 receptor chain *Il4ra*, is significantly downregulated in the hearts of patients with heart failure. Next, we showed that *Il13ra1*‐deficient mice develop severe myocardial dysfunction and dyssynchrony compared to wild‐type mice (left ventricular ejection fraction 29.7±9.9 versus 45.0±8.0; *P*=0.004, left ventricular end‐diastolic diameter 4.2±0.2 versus 3.92±0.3; *P*=0.03). A bioinformatic analysis of mouse hearts indicated that interleukin‐13 receptor α1 regulates critical pathways in the heart other than the immune system, such as extracellular matrix (normalized enrichment score=1.90; false discovery rate q=0.005) and glucose metabolism (normalized enrichment score=−2.36; false discovery rate q=0). Deficiency of *Il13ra1* was associated with reduced collagen deposition under normal and pressure‐overload conditions.

**Conclusions:**

The results of our studies in humans and mice indicate, for the first time, a role of interleukin‐13 receptor α1 in myocardial homeostasis and heart failure and suggests a new therapeutic target to treat heart disease.

## Introduction

Activation of the immune system and release of proinflammatory cytokines dictate the pathophysiology of acute and chronic myocardial diseases.^1^ Proinflammatory cytokines worsen adverse cardiac remodeling and dysfunction by destructive effects on cardiomyocytes and extracellular matrix.^2^ Attempts to improve patient outcomes by inhibition of pro‐inflammatory cytokines have failed and, in some cases, have even led to exacerbation of heart failure (HF).^2^, ^3^, ^4^ Thus, there is a need to explore new immunomodulation pathways to improve HF therapy.^5^

The effects of anti‐inflammatory cytokines on the heart have been less investigated. Interleukin (IL)‐4 and IL‐13 are T‐helper type‐2 anti‐inflammatory cytokines studied extensively for their involvement in the pathogenesis of parasitic infection, asthma, and allergic diseases.^6^ However, their potential role in heart disease remains controversial. Both activation and deficiency of IL‐13 and IL‐4 have been linked to conflicting profibrotic^7^, ^8^, ^9^ and antifibrotic effects.^10^, ^11^ Given these inconsistent results, we aimed to determine the role of IL‐13 and IL‐4 signaling in the heart. Both cytokines act via the common type‐2 interleukin‐4 receptor (IL‐4R) composed of interleukin‐13 receptor α1 (IL‐13Rα1) and IL‐4Rα. Hence, deficiency in IL‐4 could be compensated by activation of IL‐13 signaling via the common type‐2 IL‐4R, and vice versa. On the other hand, specific deletion of the IL‐13Rα1 chain could prevent the effects of compensatory activation and provide a unique opportunity to study type‐2 IL‐4R signaling in the heart.^12^

Here, we provide evidence for a previously unrecognized, protective, regulatory role of IL‐13Rα1 and type‐2 IL‐4R signaling in myocardial homeostasis, metabolism, and repair. A potential implication of our study is the development of novel therapies for myocardial disease.

## Methods

### Human Heart Samples

Tissue samples were obtained from the left ventricular (LV) free wall of 177 HF patients undergoing heart transplantation (94 ischemic and 77 idiopathic dilated cardiomyopathy, 1 valvular and 5 others) and from 136 unused donor hearts, enrolled in the MAGNet consortium (http://www.med.upenn.edu/magnet/index.shtml). Gene expression analysis was done as previously described.^13^ All procedures involving human tissue were approved by the Institutional Review Board at the University of Pennsylvania and the Tel‐Hashomer Medical Center.

### Mice

*Il13ra1* ^*−/−*^ mice were generated as previously described.^14^ In these animals, disrupted *Il13ra1* contains a lacZ reporter cassette used for β‐galactosidase staining in mouse heart tissue. C57BL/6 wild‐type (WT) mice were obtained from Harlan Laboratories (Rehovot, Israel). In all experiments, age‐matched and sex‐matched mice were housed under specific pathogen‐free conditions and maintained with 12‐hour light and dark cycles, according to institutionally approved protocols of the Animal Care Committee at the Tel‐Hashomer Medical Center, Tel‐Aviv University.

### Metabolic Studies

A glucose tolerance test was carried out in *Il13ra1* ^*−/−*^ mice and their control littermates, after 16 hours of overnight fasting and an intraperitoneal injection of 2 g of glucose per kilogram body weight. Blood glucose was measured on samples obtained by tail bleeding before glucose administration and after 30, 60, 90, and 120 minutes, using a FreeStyle Optium glucose meter (Abbott Diabetes Care, Alameda, CA). For an insulin tolerance test, mice were fasted for 6 hours and injected intraperitoneally with insulin (0.75 U kg^−1^ body weight) (Eli Lilly, Indianapolis, IN), and blood glucose levels were measured before and 15, 30, 60, 90, and 120 minutes after the injection. Body composition analysis (fat mass) in mice was assessed by nuclear magnetic resonance using a Bruker Mice Minispec NMR analyzer (Bruker Optics, Billerica, MA).

### Histological Analysis

To determine *Il13ra1* gene expression in mouse heart, we used a transgenic mouse that expresses a *lacz*‐interrupted *Il13ra1* gene. Hearts were harvested, cryosectioned into 5‐μm sections, and placed onto slides. Sections were fixed with 0.125% glutaraldehyde, permeabilized with 0.01% Na‐deoxycholate and 0.02% NP‐40. A signal was detected by incubating with 1 mg/mL X‐gal at 37°C for 3 hours. Next, to visualize cardiomyocytes in the X‐gal–stained heart sections, slides were costained with antibodies against α‐cardiac actin (Santa Cruz Biotechnology, Dallas, TX, catalog number sc‐58670).

To determine IL‐13Rα1 presence in the human myocardium, a cardiac tissue biopsy was obtained from the right atrium of a 70‐year‐old HF patient during a coronary artery bypass graft surgery. The specimen was fixed in formaldehyde 4%, paraffin embedded, and sectioned into 5‐mm sections. The sections were immunostained with the primary antibodies against IL‐13Rα1 (Abcam, Cambridge, MA, catalog number ab79277) followed by incubation with peroxidase‐conjugated AffiniPure donkey antirabbit (Jackson Immunoresearch Laboratories, West Grove, PA, catalog number 711‐035‐152), according to the manufacturer's protocol. For a negative control, the same samples and protocol were used, but the primary antibody was omitted.

To analyze fibrosis and hypertrophy, hearts were harvested from 3‐month‐old *Il13ra1* ^*−/−*^ and WT mice, washed with phosphate‐buffered saline and then fixed in 4% paraformaldehyde overnight. Adjacent blocks were embedded in paraffin, sectioned into 5‐μm slices, and stained with Masson trichrome according to standard procedure. To quantify perivascular fibrosis in comparably sized coronary arteries, we photographed all arteries with a diameter of 50 to 80 μm in each slide and analyzed collagen deposition by automated image analysis using ImageJ software (http://rsbweb.nih.gov/ij/).^15^ To assess cardiomyocyte hypertrophy and cardiac fibrosis in a pressure overload model, hearts were harvested 3 weeks after transverse aortic constriction (TAC). Wheat germ agglutinin staining was used to measure cardiomyocyte diameter, and cardiac fibrotic area was evaluated after Masson trichrome staining.

### Pressure Overload Model in Mice

TAC was performed in 10‐week‐old *Il13ra1* ^*−/−*^ and WT female mice. Animals were anesthetized with 1% to 2% isoflurane in 100% oxygen delivered through a volume‐cycled rodent respirator. Midline sternotomy was performed, the aorta was exposed, and a 6.0 prolene suture was placed around the aorta distal to the brachiocephalic artery. The suture was tightened around a blunt 27‐gauge needle placed adjacent to the aorta. The needle was then removed, and the chest and overlying skin were closed with a 5‐0 absorbable suture. Mice were allowed to recover from anesthesia under warm conditions. Mortality during and immediately following the procedure was ≈10%.

### Mouse Echocardiography

Transthoracic echocardiography and speckle‐tracking strain imaging were performed with a mouse echocardiography system (Vevo 2100 Imaging System; VisualSonics, Toronto, Ontario, Canada) equipped with a 22‐ to 55‐MHz linear‐array transducer (MS250 MicroScan Transducer, VisualSonics, Toronto, Ontario, Canada).

Speckle‐tracking–based strain analysis was performed for strain quantification in the radial axes. Echocardiographic parasternal long‐axis images were acquired at a frame rate of 280 frames per second. Three consecutive cardiac cycles were selected, and their endocardium and epicardium borders were traced. Each LV image in long axis was divided into 6 segments for regional speckle‐tracking–based strain analysis: anterior base, anterior mid, anterior apex, posterior apex, posterior mid, and posterior base. Peak strain data were recorded from each segment for regional speckle‐tracking–based strain analysis. Global strain of the LV was calculated as the averaged peak strain obtained from all 6 segments.

### Western Blotting

Proteins were extracted from the hearts of *Il13ra1* ^*−/−*^ and WT mice or H9C2 cells using a RIPA buffer (Sigma‐Aldrich, St. Louis, MO) supplemented with Complete Mini, EDTA‐free, protease inhibitor cocktail (Roche Diagnostics, Indianapolis, IN, catalog number: 11 836 170 001). Following separation on an SDS‐PAGE, proteins were transferred to a nitrocellulose membrane using the iBlot Dry Blotting System (Invitrogen Corporation, Carlsbad, CA). Membranes were stained with a primary antibody overnight at 4°C, washed, and incubated with the appropriate secondary antibody for 45 to 60 minutes at room temperature. Specific reactive bands were detected using the SuperSignal West Pico Chemiluminescent Substrate (Thermo Scientific, Rockford, IL). The antibodies used were anti–signal transducer and activator of the transcription (STAT)3 (Cell Signaling Technology, Beverly, MA, catalog number 9139), anti‐STAT6 (Cell Signaling Technology, Beverly, MA, catalog number 5397), anti–phosphorylated STAT3 (Cell Signaling Technology, Beverly, MA, catalog number 9145), anti–phosphorylated STAT6 (Santa Cruz Biotechnology, Dallas, TX, catalog number sc‐11762‐R), anti‐actin (Santa Cruz Biotechnology, Dallas, TX, catalog number sc‐58670), and anti–α tubulin (Sigma‐Aldrich, Saint Louis, MO, catalog number T9026).

### IL‐13 Signaling in H9C2 Cardiomyoblast Cell Culture

H9C2 cells were cultured in DMEM medium (Biological Industries, Beit Haemek, Israel) supplemented with 10% fetal bovine serum, 1% penicillin: streptomycin (pen:strep) and 1% glutamine, at 37°C in a humidified incubator with 5% CO_2_. Cells were seeded in 6‐well plates at a concentration of 4×10^6^ cells per well and treated with IL‐13 (10 ng/mL). Protein was extracted at 0, 2 minutes, 5 minutes, 15 minutes, 30 minutes, 1 hour, 6 hours, 24 hours, and 48 hours after the addition of the cytokine, and western blot was performed for STAT6 and STAT3 signaling as described above.

### Quantitative Real‐Time PCR in Mouse Heart

Mice were euthanized, and their hearts were harvested, washed in phosphate‐buffered saline, and snap frozen in liquid nitrogen. RNA was purified from snap‐frozen hearts with an RNeasy Mini Kit (Qiagen, Valencia, CA) following the manufacturer's instructions. Reverse transcription was performed using a High Capacity cDNA Reverse Transcription Kit (Applied Biosystems, Foster City, CA). Real‐time quantitative polymerase chain reaction was performed using the glyceraldehyde 3‐phosphate dehydrogenase (*Gapdh*) as a reference gene, which showed stable levels of expression in WT and *Il13ra1* ^*−/−*^ heart samples. All reactions were run as triplicates. Reactions were performed in a total volume of 10 μL containing cDNA equivalent to 50 ng of RNA from each sample. Real‐time quantitative polymerase chain reaction was performed using an ABI StepOnePlus System (Applied Biosystems, Foster City, CA). Primers were designed with PrimerBank (http://pga.mgh.harvard.edu/primerbank/) or Primer‐BLAST (http://www.ncbi.nlm.nih.gov/tools/primer-blast/). The sequences of all the primers and probes used for real‐time quantitative polymerase chain reaction are listed in Table.

**Table 1:** Sequences of All Primers and Probes Used for Real‐Time Quantitative Polymerase Chain Reaction

Gene	Forward	Reverse
*Mmp12*	CATGAAGCGTGAGGATGTAGAC	TGGGCTAGTGTACCACCTTTG
*Thbs1*	GCAGCACACACAGAAGCATT	CAATCAGCTCTCACCAGCAG
*Stat6*	CTCTGTGGGGCCTAATTTCCA	CATCTGAACCGACCAGGAACT
*Col3a1*	CTGTAACATGGAAACTGGGGAAA	CCATAGCTGAACTGAAAACCACC
*Timp1*	CCAGAACCGCAGTGAAGAGTT	AAGCTGCAGGCACTGAGTG
*Gapdh*	TCGTCCCGTAGACAAAATGG	TTGAGGTCAATGAAGGGGTC
*Tgfb1*	TGACGTCACTGGAGTTGTACGG	GGTTCATGTCATGGATGGTGC
*Mmp9*	GGACCCGAAGCGGACATTG	CGTCGTCGAAATGGGCATCT
*Col1a1*	CGAAGGCAACAGTCGCTTCA	GGTCTTGGTGGTTTTGTATTCGAT
*Stat3a*	TaqMan probes	
*Stat3b*	TaqMan probes	

### Plasma Cytokine Levels

To measure plasma pro‐ and anti‐inflammatory cytokine levels, retro‐orbital bleeding was performed on 10‐week old *Il13ra1* ^*−/−*^ and WT mice under light isoflurane anesthesia. Whole blood was collected into plasma collecting tubes (MiniCollect Tube, Greiner Bio‐One, Monroe, NC) and placed on ice. Samples were then centrifuged for 10 minutes at 2000*g*. Cytokine plasma concentrations were measured using the commercially available mouse Luminex Multiplex Platform (R&D Systems, Minneapolis, MN) and enzyme‐linked immunosorbent assay (BioLegend, San Diego, CA). All procedures where performed according to the manufacturer's instructions.

### Gene Arrays and Bioinformatic Analysis

To search for differentially regulated gene networks in the absence of the *Il13ra1* gene, we performed a comprehensive gene analysis by hybridizing microarray chips with RNA probes prepared from mouse *Il13ra1* ^*−/−*^ and WT hearts. Briefly, total RNA was extracted from hearts from 12‐week‐old *Il13ra1* ^*−/−*^ and WT male mice, using EZ RNA (Biological Industries, Beit Haemek, Israel) according to the manufacturer's instructions. Total RNA was quantified by using a spectrophotometer and confirmed by Qubit Fluorometric Quantitation (Life Technologies, Grand Island, NY). Gene arrays were performed using the Affymetrix Mouse Gene 2.0 ST Array (Affymetrix, Santa Clara, CA) and were robust multi‐array average‐normalized.^16^ Differentially expressed genes were found by fitting linear models and computing empirically moderated t‐statistics as implemented in the R/bioconductor limma package^17^ with a Benjamini‐Hochberg adjusted *P*‐value cutoff of 5%.

Gene set enrichment analysis (GSEA) was based on software provided by the Broad Institute of MIT and Harvard.^18^ To retain statistical power we limited the analysis to 171 gene sets that represented *Kyoto Encyclopedia of Genes and Genomes* pathways. The sets were obtained from the MSigDB v.4.0 database (http://www.broadinstitute.org/gsea/msigdb/index.jsp) and included all the gene sets in the *CP: Kyoto Encyclopedia of Genes and Genomes* collection, which has between 15 and 500 genes (these cutoffs are the default ones used by the Broad Institute's GSEA software). We retained all the default parameters except that the null model was based on gene set randomization to assess statistical significance, rather than on the default phenotype randomization, in order to accommodate the small sample size.

### Constraint‐Based Modeling of Metabolism

A metabolic network consisting of m metabolites and n reactions can be represented by an m×n stoichiometric matrix S, where m is the number of metabolites, n is the number of reactions, and the entry S_ij_ represents the stoichiometric coefficient of metabolite i in reaction j. A genome‐scale metabolic model imposes mass balance, directionality, and flux capacity constraints on the space of possible fluxes in the reactions of the metabolic network through a set of linear equations:

(1) S×V=0

(2) $$V_{\min}<V<V_{\max}$$

V stands for the flux vector for all the reactions in the model. The exchange of metabolites with the environment is represented as a set of reactions, enabling a predefined set of metabolites to be either taken up or secreted from the tissue. The steady‐state assumption represented in Equation (1) constrains the production rate of each metabolite, making it equal to its consumption rate. Enzymatic directionality and flux capacity constraints define lower and upper bounds on the fluxes and are embedded in Equation (2). Flux vectors satisfying these conditions are referred to as feasible steady‐state flux distributions.

### Integrative Metabolic Analysis Tool

We used a standard reconstruction of the human metabolic network because it is thought to be close enough to the murine network but more comprehensive than the available mouse models.^19^ In each sample, gene expression levels were discretized and classified according to the following 3 levels: high (top 25%), low (bottom 25%), or moderate (the remaining 50%). We then defined the metabolic state of the 2 conditions of interest (WT versus *Il13ra1‐*deficient) by combining the samples for each of the conditions. A gene was considered highly expressed if it had been highly expressed in two‐thirds of the samples of the state, and similarly for lowly expressed genes. The integrative metabolic analysis tool (iMAT) analysis translates the metabolic state inferred from gene expression into additional constraints in the metabolic model. It then predicts a feasible solution space for the specific condition by solving a mixed integer linear program that finds a steady‐state flux distribution satisfying stoichiometric and thermodynamic constraints while maximizing the number of reactions whose activity is consistent with their expression.^20^ To study the metabolic phenotypes in each of the conditions, we constrained the iMAT agreement level of gene expression and reaction activity to its maximum in each of the conditions and then found the maximum and minimum activity of each reaction subject to maintaining that agreement level (ie, we conducted a flux variability analysis under the additional constraint of the agreement level). We then conducted Monte‐Carlo sampling to obtain 2000 flux vectors from the flux space defined by the reaction activity limits.

### Pathway Enrichment Analysis

We tested which functional metabolic pathways were enriched based on the iMAT‐derived sampling analysis. First, based on the median, we concluded whether a reaction was up‐ or downregulated. Second, we used a hypergeometric statistical test (a binomial statistic representing the likelihood of finding x out of K items in N drawings without replacement from a group of M objects), to conclude regulation in the pathway level. Finally, all pathways underwent a false discovery rate multiple hypothesis correction test.

### Statistical Analysis

Statistical analysis was performed with the R statistical package, version 3.2.2 (R Foundation for Statistical Computing, Vienna, Austria), for human cardiac gene expression data, and GraphPad Prism version 6.00 for Windows (GraphPad Software, San Diego, CA) for all the mouse experiments. All values are expressed as mean±SEM. Kruskal‐Wallis rank sum test was applied to compare the expression of genes in human unused donor hearts with different groups of HF. Differences between means of 2 groups were compared by Student t test or Mann‐Whitney test, where data were not normally distributed. One‐way ANOVA with Bonferroni correction was used to compare radial strain and strain rate between different heart segments. Two‐way repeated‐measures ANOVA with Bonferroni correction was used to test whether measurements of weight, glucose tolerance test, insulin tolerance test, and LV function and structure after TAC operations varied over time among the experimental groups. Differences were considered significant at a *P*<0.05.

## Results

### Expression of *Il13ra1* in the Human Heart

**Figure 1:**
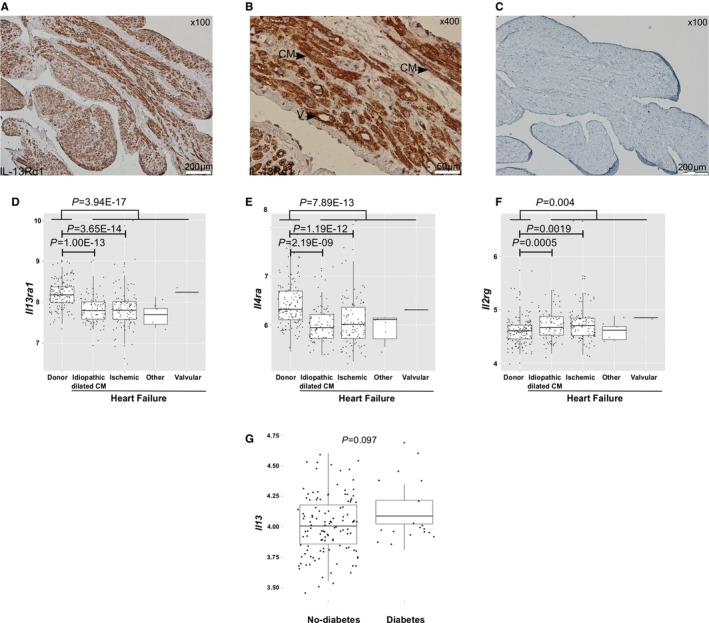
Type‐2 interleukin (IL)‐4R signaling is differentially expressed between human failing and donor hearts. A, Staining for IL‐13Rα1 in a cardiac tissue biopsy obtained from the right atrium of a 70‐year‐old heart failure patient during a coronary artery bypass graft surgery (×100). B, IL‐13Rα1 is present on cardiomyocytes in the human myocardium (×400). C, Same cardiac tissue sample excluding the primary antibody (negative control, ×100). D, Reduced *Il13ra1* expression in failing hearts (n=177) compared with unused donor hearts (n=136) (*P*=3.94×10^–17^, Kruskal‐Wallis test). E, Reduced *Il4ra* expression in failing hearts (n=177) compared with unused donor hearts (n=136) (*P*=7.89×10^–13^, Kruskal‐Wallis test). F, Overexpression of *Il2rg* in failing hearts compared to unused donor hearts (n=136) (*P*=0.004, Kruskal‐Wallis test). G, *Il13* is upregulated in donor hearts (n=136) with a history of diabetes mellitus (*P*=0.097, Mann‐Whitney test). CM indicates cardiomyocyte; LVEF, left ventricular ejection fraction; V, blood vessel.

First, we aimed to determine whether IL‐13Rα1 is present in the human heart. We obtained cardiac tissue samples from the right atrium of a patient with HF and found a robust staining for IL‐13Rα1 (Figure 1A through 1C). Next, we analyzed gene expression of tissue from the hearts of patients with end‐stage HF (n=177) and unused donor hearts (controls, n=136), obtained by the MAGNet consortium (www.med.upenn.edu/magnet). Remarkably, the expression of the chains comprising type‐2 IL‐4R, *Il13ra1* and *Il4ra* were downregulated in the failing hearts of patients with ischemic and dilated cardiomyopathy, compared with controls (Figure 1D and 1E). In contrast, the expression of the unspecific *Il2rg* subunit, common to type‐1 IL‐4R and other cytokine receptors, was upregulated in the failing hearts (Figure 1F). The expression of *Il13* and *Il13ra2* did not differ between donor and failing hearts, but *Il4* was downregulated in the failing hearts (Table S1). Notably, we found a trend for increased expression of the *Il13* gene in a subset of donor hearts with a history of diabetes mellitus (Figure 1G; Table S2). These results suggest that signaling via IL‐13Rα1 and the type‐2 IL‐4R could be implicated in the pathobiology of HF.

### Role of IL‐13Rα1 in Cardiac Structure and Function

**Figure 2:**
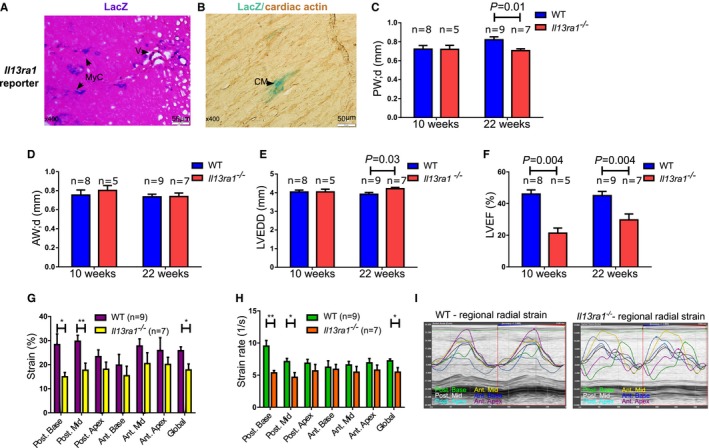
*Il13ra1* is expressed in mouse hearts and plays an important role in cardiac function and structure. A, Staining for β‐galactosidase activity for detection of lacZ reporter in the hearts of *Il13ra1* ^−/−^ mice reveals myocardial and blood vessel expression of *Il13ra1* (×400). B, Double staining for cardiac actin and β‐galactosidase activity demonstrates *Il13ra1* expression in striated cardiomyocytes (×400). Echocardiography assessment of cardiac structure and function of 10‐ and 22‐week‐old *Il13ra1* ^−/−^ and WT male mice (Student t test). C, Decreased diastolic posterior wall thickness, in 22‐week‐old *Il13ra1* ^−/−^ mice. D, No difference in diastolic anterior wall thickness between study groups. E, Increased LVEDD in 22‐week‐old *Il13ra1* ^−/−^ mice. F, Systolic dysfunction in *Il13ra1* ^−/−^ mice at 10 and 22 weeks of age. G, Reduced radial strain in *Il13ra1* ^−/−^ mice, with the main difference involving the posterior segments. H, Reduced radial strain rate in *Il13ra1* ^−/−^ mice (*P<0.05 and **P<0.01, 1‐way ANOVA). I, Representative regional strain analysis of LV movement during cardiac cycle demonstrates marked desynchrony in contractility of *Il13ra1* ^−/−^ mouse heart compared with WT. AW;d indicates anterior wall diastole; CM, cardiomyocyte; LV, left ventricle; LVEDD, left ventricular end‐diastolic diameter; LVEF, left ventricular ejection fraction; MyC, myocardial; PW;d, posterior wall diastole; V, blood vessel; WT, wild type.

To further explore the role of IL‐13Rα1 in the heart, we studied hearts of IL‐13Rα1 whole‐body knockout mice (*Il13ra1* ^*−/−*^). These mice harbor a functional deletion of type‐2 IL‐4R but have an intact type‐1 IL‐4R and therefore provide an opportunity to distinguish between the roles of type‐1 and type‐2 IL‐4R in the myocardium.^14^ We first aimed to determine whether IL‐13Rα1 is expressed in mouse hearts. Because the construction of *Il13ra1* ^*−/−*^ mice included an in‐frame insertion of a lacZ reporter gene,^14^ we used β‐galactosidase activity as a biomarker of *Il13ra1* expression. Double staining for both cardiac actin and β‐galactosidase activity revealed that *Il13ra1* is expressed by cardiomyocytes in normal mouse myocardium (Figure 2A and 2B, Figure S1).

Next, we sought to define the role of IL‐13Rα1 in the structure and function of the heart. Using a small animal echocardiography, we found a significant systolic dysfunction in *Il13ra1* ^*−/−*^ male mice at the age of 10 weeks, accompanied by mild LV dilatation and posterior wall thinning at 22 weeks (Figure 2C through 2F; Tables S3 and S4). These findings were supported by a speckle‐tracking strain analysis, a more sensitive method for assessing global and region‐specific myocardial contractility.^21^ Radial strain and strain rate, parameters of myocardial contractility, were significantly reduced in *Il13ra1* ^*−/−*^ mice compared with controls (Figure 2G and 2H). Furthermore, speckle‐tracking echocardiography revealed marked desynchronization in contractility of different segments of the LV (Figure 2I), a characteristic of advanced HF. Finally, to exclude proinflammatory cytokines as a cause of cardiac dysfunction in the mutant mice, we measured plasma cytokines in WT and *Il13ra1* ^*−/−*^ mice. Interestingly, the plasma levels of tumor necrosis factor‐α, a biomarker of inflammation, which has myogenic and antifibrotic properties,^22^ were lower in *Il13ra1* ^*−/−*^ compared with WT mice. Levels of other cytokines were similar (Figure S2). Furthermore, myocardial dysfunction in *Il13ra1* ^*−/−*^ was exclusive to male but not female mice (Figure S3). These findings indicate that *Il13ra1* deficiency is associated with significant myocardial dysfunction.

### STAT3 and STAT6 Mediate Myocardial IL‐13Rα1 Signaling in the Myocardium

**Figure 3:**
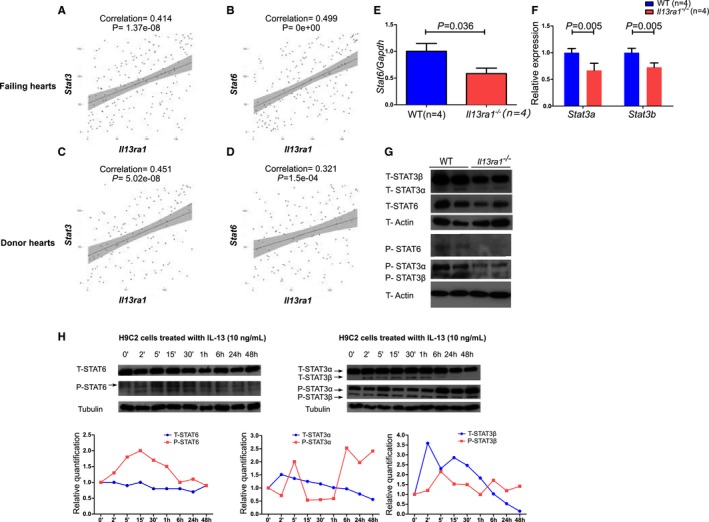
Interleukin (IL)‐13Rα1 regulates STAT3 and STAT6 signaling in human and mouse hearts. Spearman rank correlation between *Il13ra1* gene expression in failing human heart (n=177 samples) and (A) *Stat3* expression. B, *Stat6* expression. Spearman rank correlation between *Il13ra1* gene expression in human donor hearts (n=136 samples) and (C) *Stat3* expression. D, *Stat6* expression. Real‐time quantitative polymerase chain reaction from hearts of wild‐type and *Il13ra1* ^−/−^. E, Reduction in *Stat6* in mutant hearts by 41%. F, Reduction in *Stat3a* and *Stat3b* expression by 33% and 27% (Student t test; each experiment was performed in triplicate). G, western blot demonstrates a reduction in total and phosphorylated STAT3 and STAT6 proteins in *Il13ra1* ^−/−^ mouse hearts. H9C2 rat cardiomyoblasts were cultured in 6‐well plates at a concentration of 4×10^6^ cells per well and treated with IL‐13 (10 ng/mL). Protein was extracted at consecutive time points, and western blot was performed for STAT6 and STAT3 signaling. H, IL‐13 caused STAT6 phosphorylation, which peaked 15 minutes after treatment. IL‐13 increased total STAT3 α and β proteins, and caused a bimodal phosphorylation of STAT3 α and β with an early (5 minutes) and late (6 hours) activation. P indicates phosphorylated; T, total.

Because of the sex‐specific effects of *Il13ra1* deficiency on mouse phenotype, we focused our further studies on male mice. Cytokines and their receptors exert their transcriptional modifications via activation of the STAT family of genes. Particularly, STAT3 and STAT6 are implicated in IL‐13/IL‐4 signaling in several cell lines.^23^, ^24^ Significantly, our human heart data indicated that *Il13ra1* is correlated with *Stat3* and *Stat6* gene expression (Figure 3A through 3D). To confirm these findings, we analyzed gene expression and proteins from hearts of WT and *Il13ra1* ^*−/−*^ mice. Indeed, *Stat3a*,* Stat3b*, and *Stat6* gene expression were downregulated in *Il13ra1* ^−/−^ male mice (Figure 3E and 3F). Western blot analysis of heart lysates indicated fewer total and phosphorylated STAT3 and STAT6 proteins in the hearts of *Il13ra1* ^*−/−*^ than in WT mice (Figure 3G).

To confirm these findings and localize them to cardiomyocytes, we stimulated cultured rat cardiomyoblasts (H9C2 cell line) with IL‐13 cytokine (10 ng/mL) and demonstrated an increase in STAT6 and STAT3α and β phosphorylation and total STAT3α and β protein (Figure 3H). Our findings suggest that STAT3 and STAT6 mediate IL‐13Rα1 signaling in the myocardium. These results are important because STAT3 and to a lesser extent STAT6 play an important role in cardiac homeostasis, modulating cell‐to‐cell signaling among the different components of the myocardium and regulating myocardial repair.^25^

### Pathway Analysis of IL‐13Rα1‐Dependent Gene Regulation in the Mouse Heart

**Figure 4:**
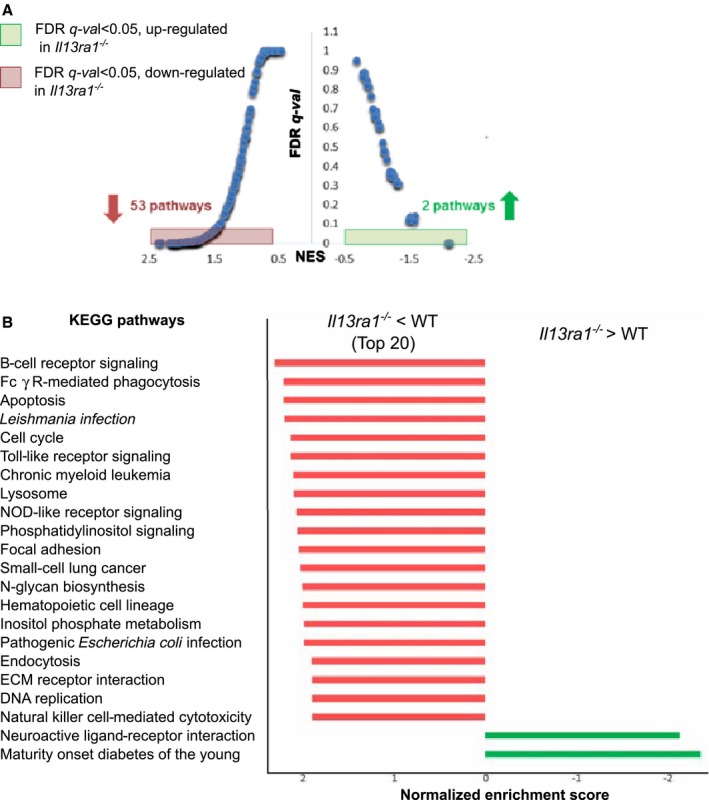
Cardiac gene‐array analysis of biological pathways targeted by interleukin (IL)‐13Rα1 in the heart. A, GSEA reveals altered biological pathways and processes based on predefined KEGG gene sets. Distribution of all gene sets based on NES and FDR q‐values corresponding to these scores. B, Data show enriched KEGG gene sets upregulated in *Il13ra1* ^−/−^ mice hearts (green) and 20 most enriched KEGG gene sets downregulated in *Il13ra1* ^−/−^ mouse hearts (red). FDR indicates false discovery rate; GSEA, gene set enrichment analysis; KEGG, *Kyoto Encyclopedia of Genes and Genomes;* NES, normalized enrichment score.

To gain further insight into the mechanism underlying LV dysfunction in *Il13ra1* ^*−/−*^ mice, we performed Affymetrix gene array from RNA purified from hearts of WT and *Il13ra1* ^*−/−*^ mice. A total of 549 genes were differentially expressed in *Il13ra1* ^*−/−*^ compared with WT hearts (empirical Bayes moderated t test; BH‐adjusted *P*<0.05). Immune function and cell adhesion genes were significantly enriched with GO terms (data not shown). To understand the causes underlying the *Il13ra1* ^*−/−*^ phenotype, we conducted GSEA of the microarray data. Briefly, GSEA tests whether the definition of gene sets, based on external biological knowledge (eg, known pathways), is collectively up‐ or downregulated with respect to a given phenotype.^18^ Here, we tested the differential expression of 171 gene sets based on *Kyoto Encyclopedia of Genes and Genomes* pathways and found that 55 pathways were differentially expressed between *Il13ra1* ^*−/−*^ and WT mouse hearts (false discovery rate q<0.05). Of these, 53 pathways were significantly downregulated in *Il13ra1*‐deficient mice hearts, and 2 were significantly upregulated (Figure 4A). As expected, many of the downregulated gene sets in the *Il13ra1*‐deficient hearts were related to inflammation and immune response. However, we found downregulated pathways that were previously unknown to be controlled by IL‐13Rα1 in the heart. These new pathways included extracellular matrix (ECM) receptor interaction, cell cycle, lysosome, focal adhesion, and apoptosis. The 2 pathways that were significantly enriched in the mutant hearts were maturity‐onset diabetes mellitus of the young and neuroactive ligand‐receptor interaction (Figure 4B). Thus, our bioinformatic analysis suggests that IL‐13Rα1 regulates important pathways in the heart other than the immune system, such as ECM and glucose metabolism.

### Reduced ECM Deposition in the Hearts of *Il13ra1* ^*−/−*^ Mice

**Figure 5:**
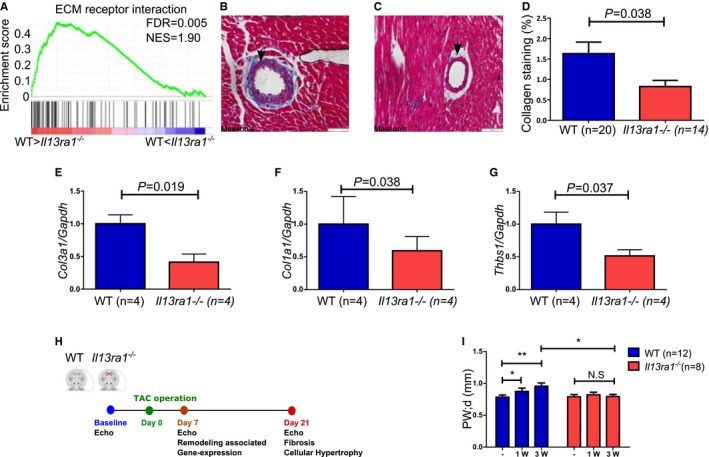
Interleukin (IL)‐13Rα1 regulates cardiac extracellular matrix deposition under homeostasis. A, GSEA of KEGG ECM receptor interaction pathway is enriched with genes that are downregulated in *Il13ra1* ^−/−^ (n=3) compared to WT (n=4) hearts. The bars represent genes included in the pathway, which are sorted by their differential expression from the most down‐regulated in *Il13ra1* ^−/−^ (left) to the most up‐regulated ones (right). The green curve corresponds to the GSEA enrichment score. B, Representative perivascular collagen deposition in WT heart (Masson trichrome). C, Representative perivascular collagen deposition in an *Il13ra1* ^−/−^ heart (Masson trichrome). D, Reduced perivascular collagen deposition in *Il13ra1* ^−/−^ hearts compared to WT (Student t test). E, Real‐time quantitative polymerase chain reaction from hearts of WT and *Il13ra1* ^−/−^. Reduced *Col3a1* expression in *Il13ra1* ^−/−^ hearts. F, Reduced *Col1a1* expression in *Il13ra1* ^−/−^ hearts. G, Reduced *Thbs1* expression in *Il13ra1* ^−/−^ hearts (Student t test; each experiment was performed in triplicate). H, Schematic outline of the TAC model protocol performed. I, Reduced posterior wall hypertrophy 3 weeks after TAC in *Il13ra1* ^−/−^ females as assessed by echocardiography (*P<0.05 and **P<0.01, 2‐way ANOVA). ECM indicates extracellular matrix; FDR, false discovery rate; GSEA, gene set enrichment analysis; KEGG, *Kyoto Encyclopedia of Genes and Genomes;* NES, normalized enrichment score; TAC, transverse aortic constriction; WT, wild type.

The GSEA of microarray data suggests that IL‐13Rα1 regulates cardiac ECM (Figure 5A), which is important for LV structural integrity, provides a scaffold for myocardial cells, and regulates myocardial function. Furthermore, the ECM components control communication among myocardial cells and are critical for myocardial repair and regeneration. Indeed, loss of ECM leads to disruption of LV structure, cardiomyocyte slippage, adverse cardiac dilatation, and HF.^26^ On the other hand, uncontrolled accumulation of ECM, ie, fibrosis, facilitates myocardial stiffness and results in diastolic dysfunction and HF.^27^ Staining WT and *Il13ra1* ^−/−^ hearts revealed a significant decrease in perivascular collagen in the hearts of *Il13ra1* ^−/−^ mice (Figure 5B through 5D), which was consistent with reduced expression of myocardial collagens (collagens I, III) and thrombospondin‐1 (Figure 5E through 5G). Together, our results indicate that IL‐13Rα1 regulates cardiac ECM, and that *Il13ra1* deficiency is associated with reduced myocardial ECM.

**Figure 6:**
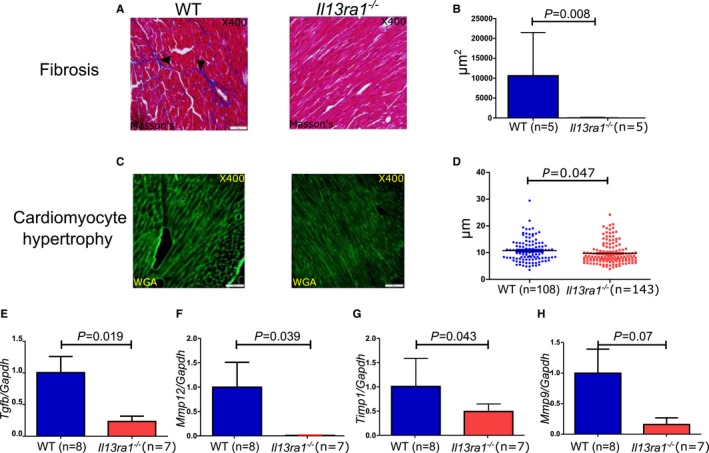
Interleukin (IL)‐13Rα1 regulates cardiac fibrosis under pathological conditions. A, Representative images of cardiac fibrosis in WT and *Il13ra1* ^−/−^ hearts 3 weeks after TAC. B, Reduced cardiac fibrotic area in *Il13ra1* ^−/−^ compared to WT hearts 3 weeks after TAC (Mann‐Whitney rank sum test). C, Representative images of cardiomyocyte hypertrophy in WT and *Il13ra1* ^−/−^ hearts 3 weeks after TAC (WGA). D, Reduced cardiomyocyte hypertrophy in *Il13ra1* ^−/−^ compared to WT hearts 3 weeks after TAC (Student t test). Reduced gene expression of key cardiac remodeling‐associated genes in *Il13ra1* ^−/−^ compared to WT hearts after TAC. E, *Tgfb*. F, *Mmp12*. G, *Timp1*. H, *Mmp9* (Student t test; each experiment was performed in triplicate). TAC indicates transverse aortic constriction; WGA, wheat germ agglutinin; WT, wild type.

Next, we sought to determine the role of IL‐13Rα1 in cardiac fibrosis. Pressure overload exposes the myocardium to significant stress, which is characterized by cardiomyocyte hypertrophy, fibrosis, apoptosis, and adverse LV remodeling.^28^ To simulate the effect of pressure overload, we induced TAC in 10‐week‐old female *Il13ra1*‐deficient and WT mice (Figure 5H). The selection of female mice was based on our finding that *Il13ra1* ^−/−^ leads to spontaneous LV dysfunction in male but not in female mice. Three weeks after TAC, *Il13ra1* ^−/−^ female mice developed less posterior wall hypertrophy compared with WT controls (Figure 5I), with no difference in cardiac systolic function (Table S5). Significantly, staining the hearts for collagen deposition revealed that the *Il13ra1* ^−/−^ myocardium was resistant to fibrosis (Figure 6A and 6B). Moreover, *Il13ra1* ^−/−^ displayed slightly less cardiomyocyte hypertrophy (Figure 6C and 6D), suggesting that the main protective effect against cardiac hypertrophy was mediated by inhibition of ECM deposition. These structural differences in the *Il13ra1* ^−/−^ hearts were accompanied by a reduction in the expression of genes regulating fibrosis and hypertrophy, including *transforming growth factor* β (*Tgfb*), *matrix metalloproteinase* (*Mmp*) *12*,* tissue inhibitor of metalloproteinase* (*Timp*) *1* and *Mmp9* (Figure 6E through 6H). Consistent with these findings in mice, we found a positive correlation between *Il13ra1* and *Tgfb* and *Timp1* expression in failing human hearts (Figure S4). Overall, our results indicate that *Il13ra1* deficiency in female mice reduces fibrosis and hypertrophy during pressure overload.

### *Il13ra1* Deficiency Leads to Metabolic Abnormalities

**Figure 7:**
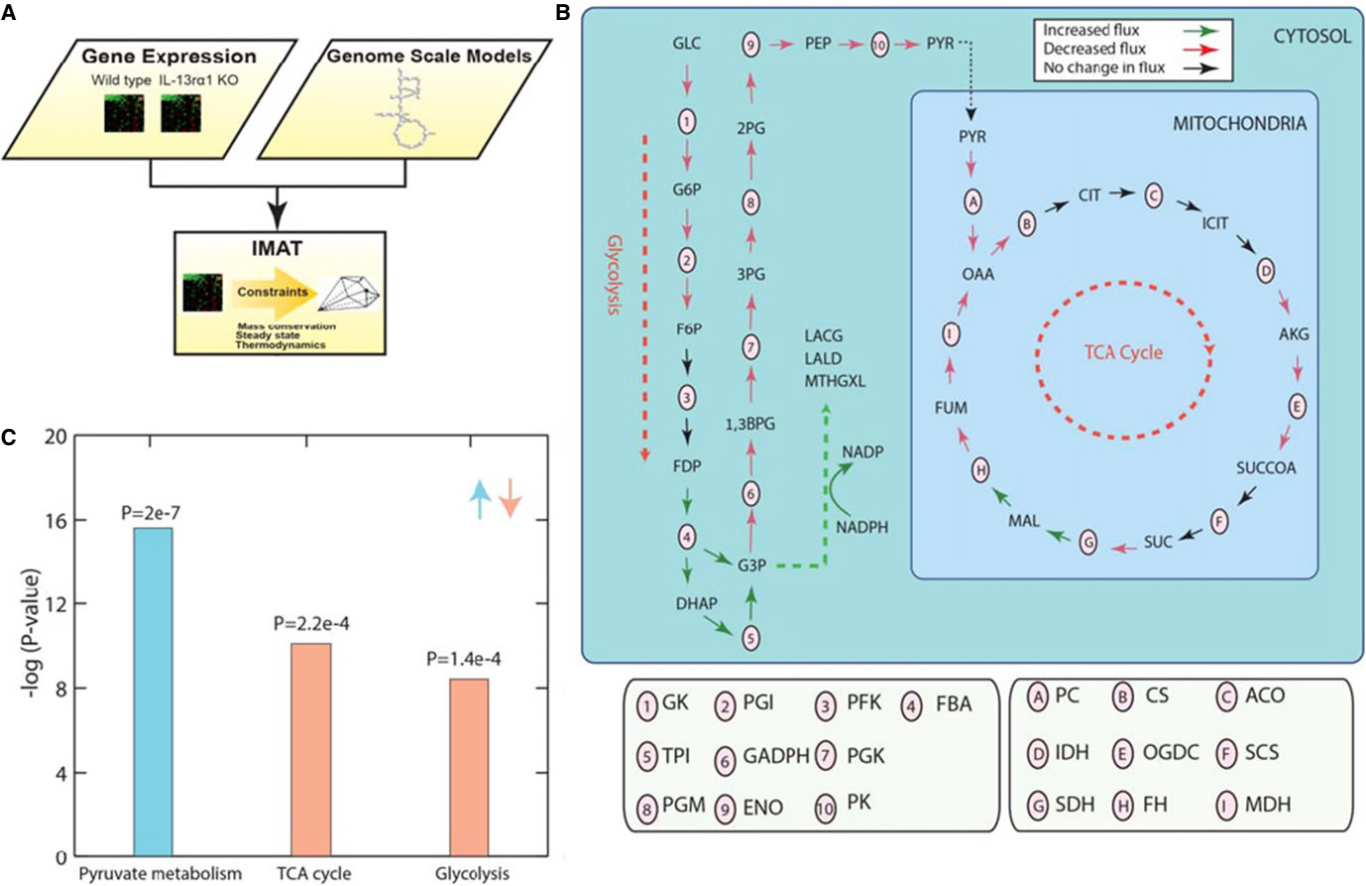
Interleukin (IL)‐13Rα1 in myocardial metabolism. A, Schematic description of the input used to simulate cardiac *Il13ra1* ^−/−^ metabolism using GSMM. High‐throughput data from cardiac tissue of wild‐type (n=4) and *Il13ra1* ^−/−^ (n=3) mice, and a GSMM were used as inputs. The iMAT algorithm integrates the gene‐expression data with a genome scale model in order to find a feasible solution space for metabolic flux distribution, thus enabling description of posttranscriptional modifications not shown by gene expression alone. B, Pathway enrichment analysis over the set of common reactions upregulated or downregulated in *Il13ra1* ^−/−^ cardiac metabolism, computed via hypergeometric test and corrected for multiple hypotheses using a false discovery rate <0.05. C, Guided by changes in gene‐expression iMAT presents an aberrant energy metabolism in *Il13ra1*‐deficient hearts, consisting of a decrease in glycolysis and tricarboxylic acid cycle, with the upregulation of pyruvate. GSMM, genome scale metabolic modeling; iMAT, integrative metabolic analysis tool; TCA cycle, tricarboxylic acid cycle. Glycolytic metabolites: GLC, glucose; G6P, glucose 6‐phosphate; F6P, fructose 6‐phosphate; FDP, fructose 1,6‐bisphosphate; DHAP, dihydroxyacetone phosphate; G3P, glyceraldehyde 3‐phosphate; 1,3BPG, 1,3 bisphosphoglycerate; 3PG, 3‐phosphoglycerate; 2PG, 2‐phosphoglycerate; PEP, phosphoenolpyruvate; PYR, pyruvate. TCA cycle metabolites: CIT, citrate; ICIT, isocitrate; AKG, α‐ketoglutarate; SUCCOA, succinyl‐CoA; SUC, succinate, FUM, fumarate; MAL, malate; OAA, oxaloacetate. Advance glycosylation end products: MTHGXL, methylglyoxal; LACG, lactoylglutathione; LALD, lactaldehyde.

Both our human gene expression analysis and the enrichment of Maturity‐onset diabetes mellitus of the young in GSEA of *Il13ra1* ^−/−^ mice hearts, suggest a possible link between IL‐13Rα1 signaling and diabetic hearts. Indeed, *Il13ra1* ^−/−^ mice displayed several systemic metabolic abnormalities including increased weight and fat gain, as well as mild abnormalities in glucose metabolism (Figure S5). To determine the effect of IL‐13Rα1 on cardiac metabolism, we analyzed the data obtained from *Il13ra1* ^−/−^ and WT mice cardiac gene arrays using genome scale metabolic modeling, which is a constraint‐based computational approach that has been widely used to study human metabolism in health and in disease.^29^ We utilized iMAT, which integrates gene expression levels measured under different conditions to predict the most likely distribution of metabolic enzyme fluxes. Importantly, iMAT can be used to predict not only the activity of a certain metabolic reaction and its direction but also (within a genome scale metabolic context) all posttranscriptional properties not accessible by gene expression alone.^20^ Pathway enrichment analysis revealed a significant downregulation of genes related to metabolic reactions associated with glycolysis, the tricarboxylic acid cycle, and upregulation of genes of the pyruvate metabolism pathway in the hearts of *Il13ra1*‐deficient mice. More specifically, we found an increase in the core reactions of pyruvate metabolism involved in the production of advanced glycosylation end products such as methylglyoxal, lactoylglutathione, and lactaldehyde (Figure 7A through 7C; Tables S6 through S8). These advanced glycosylation end products are increased in the plasma and tissues of diabetic patients and are believed to contribute to micro‐ and macrovascular complications by promoting cellular apoptosis, inflammation, and ECM crosslinking and expression.^30^ Thus, our results indicate that *Il13ra1* deficiency dysregulates glucose metabolism in the heart.

## Discussion

Our study provides new evidence that IL‐13Rα1 signaling plays an essential regulatory role in myocardial homeostasis under physiological and pathological conditions. We demonstrated the pivotal role of IL‐13Rα1 in the heart by detailed bioinformatic analysis of myocardial samples from patients with and without HF and correlated them with *Il13ra1*‐deficient mice. We demonstrated that IL‐13Rα1 is linked to pathways associated with ECM deposition and glucose metabolism. *Il13ra1* and *Il4ra* genes are downregulated in the hearts of human patients with end‐stage HF. *Il13ra1* deficiency in male mice causes marked LV dysfunction, which is associated with impaired deposition of myocardial ECM and abnormal glucose metabolism. We showed that IL‐13Rα1 regulates downstream activation of STAT3 and STAT6 in cardiomyocytes and that its deficiency attenuates cardiac fibrosis in TAC‐operated female mice. Together, our results suggest that the immune system, by IL‐13Rα1 signaling, plays a key role in cardiac homeostasis and failure in humans and mice.

The role of IL‐4 and IL‐13 signaling in myocardial disease has been controversial. IL‐4 and IL‐13 have been shown to exert powerful profibrotic effects within the heart, liver, intestines, and lungs^7^, ^8^, ^9^ and have been implicated in various chronic fibrotic diseases.^31^ In contrast, *Il13*‐deficient male mice display increased cardiac fibrosis in models of myocarditis and myocardial infarction.^10^, ^11^ Based on our findings, it may now be possible to resolve these seemingly contradictory findings. Because IL‐4 and IL‐13 have overlapping signaling via type‐2 IL‐4R, deletion of 1 cytokine may lead to increased signaling by the other cytokine and to a paradoxical increase in fibrosis and tissue damage. Indeed, *Il13*‐deficient mice with myocarditis had increased levels of IL‐4.^10^ On the other hand, the *Il13ra1*‐deficient mouse, which we used in our experiments, does not respond to either IL‐4 or IL‐13 via the type‐2 IL‐4R. Thus, we were able to demonstrate that type‐2 IL‐4R signaling plays a pivotal protective role in the myocardium. Interestingly, *Il13ra1* deficiency in female mice reduces fibrosis during pressure overload. The significance of this observation is unclear. Although fibrosis and scar formation are essential responses to acute myocardial injury, uncontrolled fibrosis may lead to adverse cardiac remodeling and HF.^27^

Our results also support the paradigm of STAT3 as a key mediator of myocardial structure and function regulating cardiac ECM deposition and hypertrophy. Similar to our findings in *Il13ra1*‐deficient mice, earlier studies have shown that STAT3 has sex‐specific effects in the myocardium. Male but not female cardiomyocyte‐restricted STAT3‐deficient mice develop impaired cardiac function, ventricular remodeling and dilatation with advancing age.^32^, ^33^ Moreover, cardiomyocyte STAT3 inhibition resulted in decreased collagen synthesis in cultured cardiac fibroblasts and attenuated pressure overload–induced cardiac fibrosis and hypertrophy.^34^ Overall, STAT3 seems to be a key mediator of IL‐13Rα1 signaling in the heart.

Based on our findings and previous reports,^6^, ^35^, ^36^ we suggest a dual role for IL‐13Rα1 in both myocardial homeostasis and repair. IL‐13Rα1 signaling is critical for cardiac ECM integrity under physiological conditions, but continuous, uncontrolled stimulation of IL‐13Rα1/STAT3 signaling during chronic cardiac stress is maladaptive and may induce excessive ECM accumulation, cardiac fibrosis and HF. Our findings are in line with previous reports, suggesting that IL‐13 signaling has healing, reparative effects on tissues, such as skeletal muscle and the lungs.^37^, ^38^

In the present report, we show that IL‐13Rα1–deficient mice exhibited mild metabolic abnormalities and altered myocardial energy metabolism consistent with the diabetic heart. However, these mice are protected from myocardial fibrosis, which is often associated with diabetic cardiomyopathy. The effect of immune stimuli on metabolic pathways has recently been recognized.^39^ For example, stimulation of macrophages with IL‐4 can induce oxidative phosphorylation and M2 polarization, whereas activation of cells through pattern recognition receptors such as Toll‐like receptor 4 (TLR4) induces HIF1α expression and promotes glycolysis, and M1 polarization.^39^ Inflammatory macrophages (M1) use glycolysis, the tricarboxylic acid cycle, the pentose phosphate pathway, fatty acid synthesis, and amino acid metabolism to proliferate and to support the production of inflammatory cytokines.^39^ M2 macrophages, which exhibit a more anti‐inflammatory phenotype, use the tricarboxylic acid cycle, fatty acid oxidation and arginine flux into the arginase pathway.^39^ Our results are complementary to recent publications showing that IL‐13 plays an important role in glucose metabolism in skeletal muscle cells and the liver. Jiang et al showed that IL‐13 increases glucose oxidation in skeletal muscle myotubes from diabetic and nondiabetic patients.^40^ IL‐13 deficiency in mice leads to increased weight gain, hyperglycemia, and hepatic insulin resistance due to the dysregulation of the IL‐13Rα1/STAT3 axis in hepatocytes.^41^ Together, our findings could be relevant for the development of new therapies for diabetic cardiomyopathy.

We are aware of several limitations in our work. First, we analyzed human cardiac biopsies, which contained various myocardial cells such as cardiomyocytes, fibroblasts, and endothelial cells. Thus, our findings cannot be solely attributed to a deficiency of the receptor in cardiomyocytes. It is possible that loss of *Il13ra1* in fibroblasts also plays a role in the development of cardiac dysfunction. Further studies using cell‐specific *Il13ra1* deletion are required to localize our results to a specific cell type. Second, to describe the effect of IL‐13Rα1 on cardiac metabolic pathways, we used a constraint‐based model, a widely used computational approach for studying metabolism on a genome scale, which has been implicated in various tissues and conditions.^20^, ^42^, ^43^ A validation at the protein level could strengthen our new findings. Third, to generate *Il13ra1* ^−/−^ mice, the *Il13ra1* gene was replaced by a cassette, which consists of a β‐galactosidase enzyme gene and a neomycin resistance gene.^14^ The construct deletes amino acids 15 824 through 22 414 of IL‐13R1 contained in exons 2 to 4 of the gene. Although replacement of the endogenous gene at a given locus can theoretically alter transcriptional patterns in any transgenic mouse model, this unwanted effect has not been described in previous publications using the *Il13ra1*‐deletion/lacz reporter mouse model.^38^, ^44^, ^45^, ^46^

In summary, our human data, together with results observed from mouse studies, provide new insights into the pleiotropic roles of IL‐13Rα1 in the heart during normal conditions and stress. A deeper understanding of the role of IL‐13Rα1 in heart disease may ultimately pave the way for the development of effective treatment for adverse heart remodeling and failure.

## Sources of Funding

This project was partially supported by a grant from the Israeli National Nanotechnology Initiative and Helmsley Charitable Trust for a focal technology area on Nanomedicines for Personalized Theranostics (Leor and Blum).

## Disclosures

None.

## Supporting information


**Table S1.** Differential Expression Analysis of IL‐13/IL‐4 Cytokines and Receptor Subunits in Failing (n=177) and Donor Human Hearts (n=136) Obtained From the MAGNet Consortium
**Table S2.** Differential Expression Analysis of IL‐13/IL‐4 Cytokines and Receptor Subunits in Donor Hearts With a History of Diabetes Obtained From the MAGNet Consortium
**Table S3.** Echocardiography Assessment of Cardiac Structure and Function of 10‐Week‐Old *Il13ra1*‐Deficient and Wild‐Type Male Mice
**Table S4.** Echocardiography Assessment of Cardiac Structure and Function of 22‐Week‐Old *Il13ra1*‐Deficient and Wild‐Type Male Mice
**Table S5.** Echocardiography Assessment of Cardiac Structure and Function of *Il13ra1*‐Deficient and Wild‐Type Female Mice After Transverse Aortic Constriction
**Table S6.** Glycolysis Cycle Reactions Change in *Il‐13ra1* ^−/−^ Hearts Compared to Wild‐Type Controls, According to Integrative Metabolic Analysis Tool Increased Flux: “↑”, Decreased Flux: “↓”
**Table S7.** Tricarboxylic Acid Cycle Reactions Change in *Il‐13ra1* ^−/−^ Hearts Compared to Wild‐Type Controls, According to Integrative Metabolic Analysis Tool Increased Flux: “↑”, Decreased Flux: “↓”
**Table S8.** Pyruvate Reactions Change in *Il‐13ra1* ^−/−^ Hearts Compared to Wild‐Type Controls, According to Integrative Metabolic Analysis Tool Increased Flux: “↑”, Decreased Flux: “↓”
**Figure S1.** Staining for β‐galactosidase activity for detection of lacZ reporter in the hearts of *Il13ra1* ^−/−^ mice, showing that the *Il13ra1* gene is expressed in all parts of mice myocardium with no visible differences among base, middle, and apex sections.
**Figure S2.** Reduced plasma tumor necrosis factor in *Il13ra1*‐deficient mice.
**Figure S3.** No difference in cardiac structure and function between 10‐week‐old *Il13ra1* ^−/−^ and wild‐type female mice as assessed by echocardiography.
**Figure S4.** Spearman rank correlation between *Il13ra1* gene expression and cardiac remodeling‐associated genes in human failing hearts (n=177) obtained from the MAGNet consortium.
**Figure S5.** *Il13ra1* ^−/−^ mice display metabolic abnormalities.
