# Supplementary material for: New Role for Interleukin‐13 Receptor α1 in Myocardial Homeostasis and Heart Failure
Source: J Am Heart Assoc. 2017 May 20;6(5):e005108. doi: 10.1161/JAHA.116.005108 (PMC5524075; doi:10.1161/JAHA.116.005108)
Supplement: Supplementary file 1 — Table S1. Differential Expression Analysis of IL‐13/IL‐4 Cytokines and Receptor Subunits in Failing (n=177) and Donor Human Hearts (n=136) Obtained From the MAGNet Consortium Table S2. Differential Expression Analysis of IL‐13/IL‐4 Cytokines and Receptor Subunits in Donor Hearts With a History of Diabetes Obtained From the MAGNet Consortium Table S3. Echocardiography Assessment of Cardiac Structure and Function of 10‐Week‐Old Il13ra1‐Deficient and Wild‐Type Male Mice Table S4. Echocardiography Assessment of Cardiac Structure and Function of 22‐Week‐Old Il13ra1‐Deficient and Wild‐Type Male Mice Table S5. Echocardiography Assessment of Cardiac Structure and Function of Il13ra1‐Deficient and Wild‐Type Female Mice After Transverse Aortic Constriction Table S6. Glycolysis Cycle Reactions Change in Il‐13ra1 −/− Hearts Compared to Wild‐Type Controls, According to Integrative Metabolic Analysis Tool Increased Flux: “↑”, Decreased Flux: “↓” Table S7. Tricarboxylic Acid Cycle Reactions Change in Il‐13ra1 −/− Hearts Compared to Wild‐Type Controls, According to Integrative Metabolic Analysis Tool Increased Flux: “↑”, Decreased Flux: “↓” Table S8. Pyruvate Reactions Change in Il‐13ra1 −/− Hearts Compared to Wild‐Type Controls, According to Integrative Metabolic Analysis Tool Increased Flux: “↑”, Decreased Flux: “↓” Figure S1. Staining for β‐galactosidase activity for detection of lacZ reporter in the hearts of Il13ra1 −/− mice, showing that the Il13ra1 gene is expressed in all parts of mice myocardium with no visible differences among base, middle, and apex sections. Figure S2. Reduced plasma tumor necrosis factor in Il13ra1‐deficient mice. Figure S3. No difference in cardiac structure and function between 10‐week‐old Il13ra1 −/− and wild‐type female mice as assessed by echocardiography. Figure S4. Spearman rank correlation between Il13ra1 gene expression and cardiac remodeling‐associated genes in human failing hearts (n=177) obtained from the MAGNet consortium. Figure S5. Il13r [file JAH3-6-e005108-s001.pdf]

SUPPLEMENTAL MATERIAL

ADDITIONAL FIGURES AND TABLES

Supplementary Figure 1.

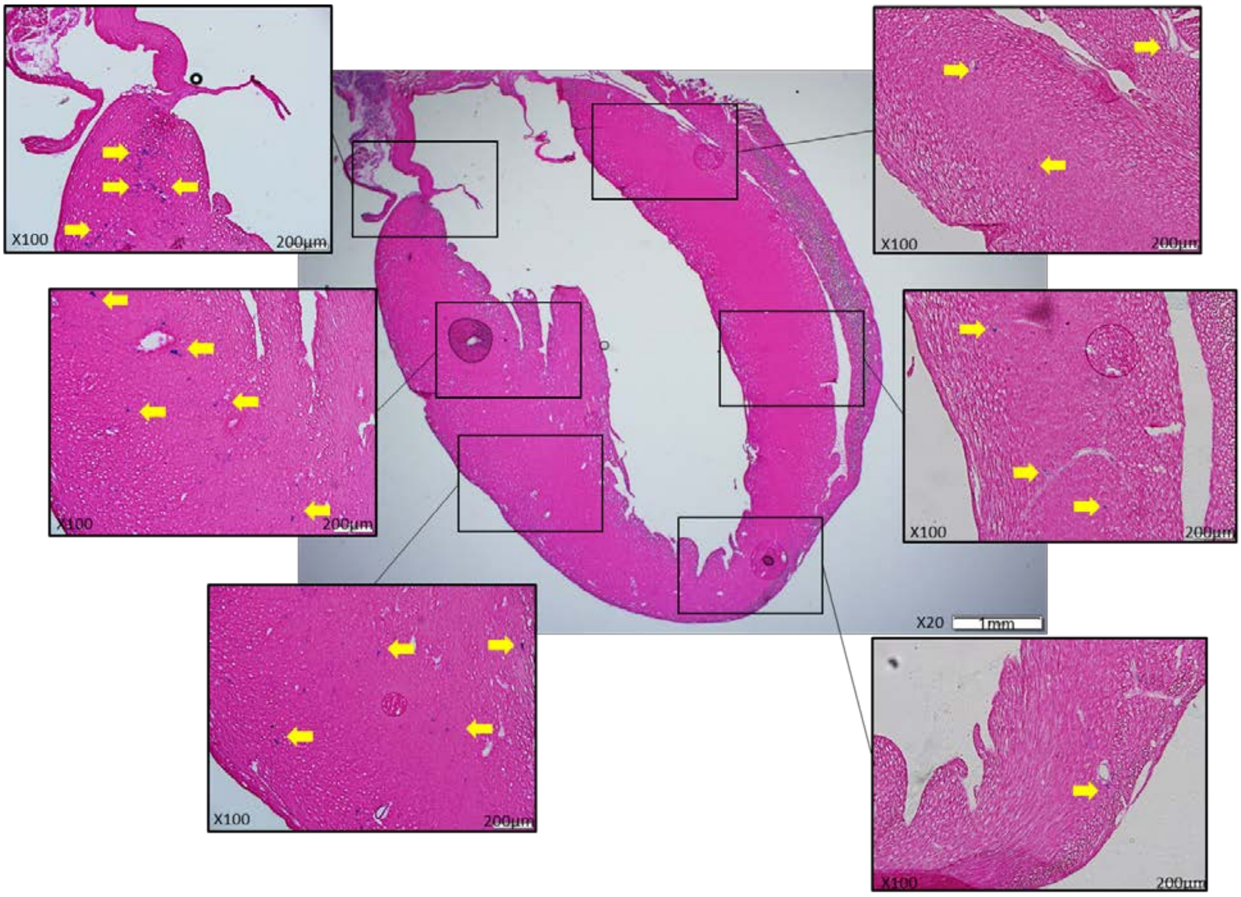

Supplementary Fig. 1. Staining for β-galactosidase activity for detection of lacZ reporter in the hearts of *Il13ra1*$^{-/-}$ mice, shows that the *Il13ra1* gene is expressed in all parts of mice myocardium with no visible differences between base/ middle/ apex sections.

Supplementary Figure 2.

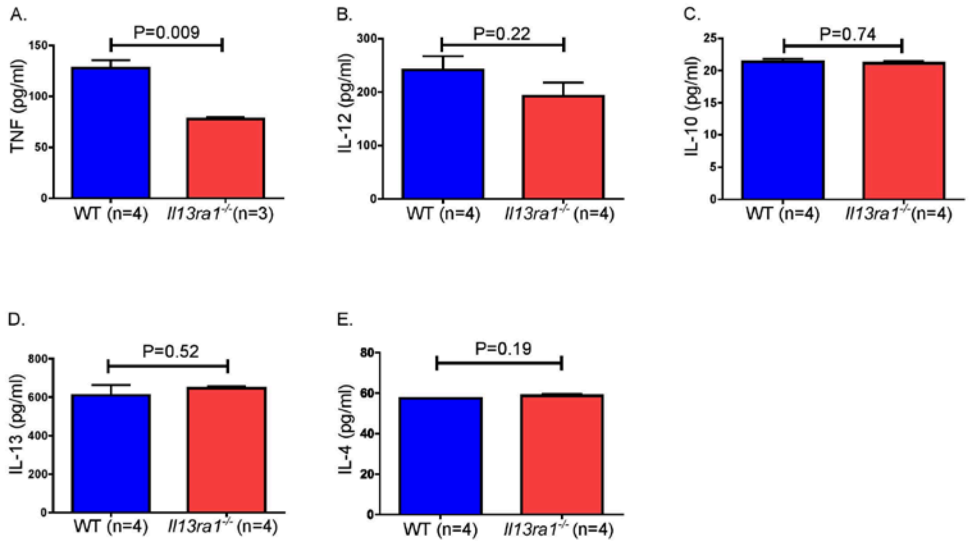

Supplementary Fig. 2. Reduced plasma tumor necrosis factor in *Il13ra1*-deficient mice. In order to rule out an indirect elevation in inflammatory cytokines as a cause for the cardiac dysfunction observed in the mutant mice we tested plasma cytokines in WT and *Il13ra1*$^{-/-}$ mice. A. TNF plasma levels were lower in *Il13ra1*$^{-/-}$ compared to WT mice. There was no difference in other inflammatory cytokines measured including B. IL-12. C. IL-10 D. IL-13 E. IL-4. Plasma IL-1β and IL-6 were not detected (Student's t-test was applied for all calculations).

Supplementary Figure 3.

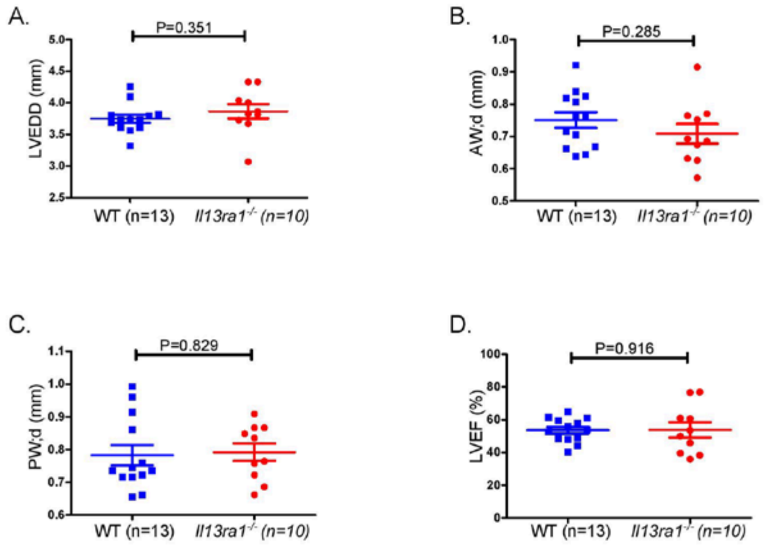

Supplementary Fig. 3. No difference in cardiac structure and function between 10- weeks old *Il13ra1*$^{-/-}$ and WT female mice as assessed by echocardiography. A. left ventricular end diastolic diameter. B. Anterior wall diameter. C. Posterior wall diameter. D. Left ventricular ejection fraction (Student's t-test).

Supplementary Figure 4.

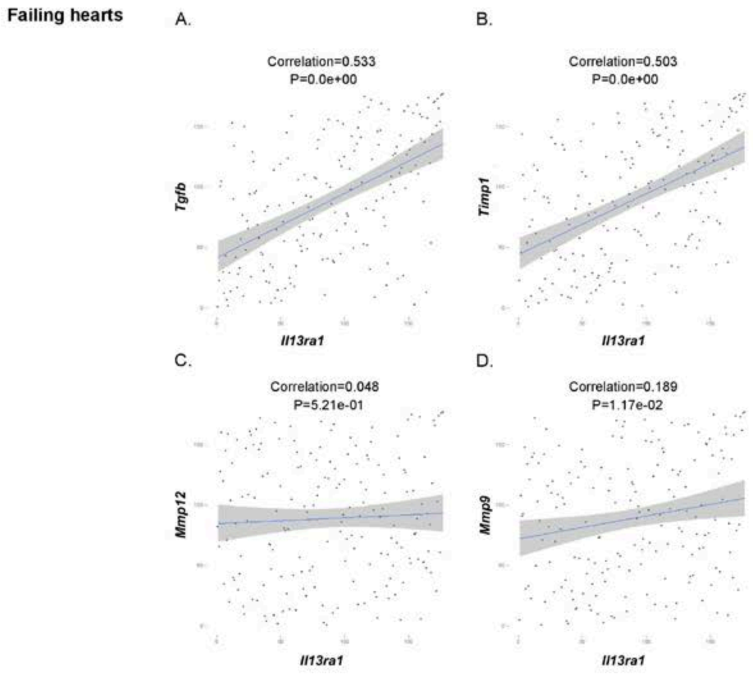

Supplementary Fig. 4. Spearman's rank correlation between *Il13ra1* gene expression and cardiac remodeling associated genes in human failing hearts (n=177) obtained from the MAGNet consortium. A. *Tgfb*. B. *Timp1*. C. *Mmp12*. D. *Mmp9*.

Supplementary Figure 5.

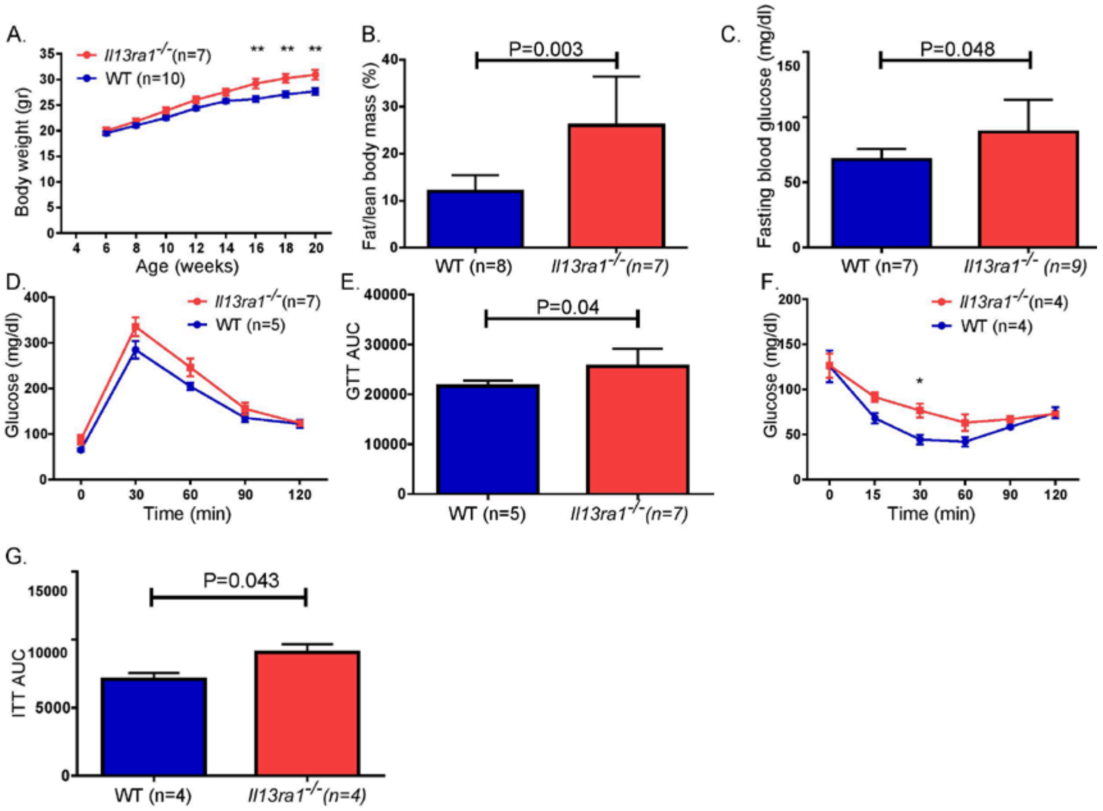

Supplementary Fig. 5. *Il13ra1$^{-/-}$* mice display metabolic abnormalities. A. *Il13ra1$^{-/-}$* gain more weight that wild-type mice. B. Higher fat/lean body mass as assessed by NMR in 22 weeks old male *Il13ra1$^{-/-}$* compared to wild-type. C. Higher fasting glucose in mutant mice. D+ E. *Il13ra1$^{-/-}$* display mildly abnormal glucose tolerance test with an elevated AUC. F+ G. *Il13ra1$^{-/-}$* display mildly abnormal insulin tolerance test with an elevated AUC. Area under the curve- AUC; Nuclear magnetic resonance- NMR. (Student's t-test was applied for all calculations, * P<0.05, **P<0.01).

<u>Supplementary Table 1.</u> Differential expression analysis of IL-13/IL-4 cytokines and receptor subunits in failing (n=177) and donor human hearts (n=136) obtained from the MAGNet consortium.

Affymetrix ID	Gene symbol	Correlation.cor	P-value	Odds ratio	Greater	Less
**X8107970**	*Il13ra1*	-0.4458	0.0000	1.2440	1.0000	0.0000
**X8173444**	*Il4ra*	-0.4074	0.0000	1.2199	1.0000	0.0000
**X8107977**	*Il2rg*	0.2057	0.0002	1.3642	0.0001	0.9999
**X8169580**	*Il4*	-0.1084	0.0555	1.2881	0.9987	0.0013
**X8174598**	*Il13ra2*	-0.0192	0.7354	1.2966	0.6545	0.3460
**X7994280**	*Il13*	0.0171	0.7628	1.3040	0.3021	0.6984

Supplementary Table 2. Differential expression analysis of IL-13/IL-4 cytokines and receptor subunits in donor hearts with a history of diabetes obtained from the MAGNet consortium.

Affymetrix ID	Gene symbol	Correlation.cor	P-value	Odds ratio	Greater	Less
**X7994280**	*Il13*	0.1439	0.0972	0.1488	0.0637	0.9371
**X8174598**	*Il13ra2*	0.1030	0.2363	0.1405	0.9320	0.0689
**X8173444**	*Il4ra*	-0.0711	0.4144	0.1430	0.7829	0.2190
**X8107970**	*Il13ra1*	-0.0678	0.4365	0.1442	0.8058	0.1961
**X8169580**	*Il4*	0.0544	0.5323	0.1464	0.2074	0.7945
**X8107977**	*Il2rg*	-0.0180	0.8366	0.1446	0.2987	0.7036

Supplementary Table 3. Echocardiography assessment of cardiac structure and function of 10 weeks old *Il13ra1*-deficient and wild-type male mice.

	Il13ra1$^{-/-}$ **(n=5)**	**Wild-type (n=8)**	**P-value**
**Anterior wall diastole (mm)**	0.80±0.11	0.75±0.15	0.545
**Anterior wall systole (mm)**	0.93±0.16	1.02±0.20	0.425
**Posterior wall diastole (mm)**	0.72±0.33	0.72±0.11	0.972
**Posterior wall systole (mm)**	0.81±0.37	1.00±0.07	0.001
**LV diastolic diameter (mm)**	4.04±0.33	4.04±0.29	0.985
**LV systolic diameter (mm)**	3.63±0.37	3.12±0.38	0.037
**LV mass (gr)**	111.39±26.96	107.99±28.39	0.83
**LV volume diastole (mm^3)**	70.53±13.43	71.73±11.70	0.87
**LV volume systole (mm^3)**	55.71±12.73	39.35±10.75	0.03
**Fractional shortening**	*9.62±3.34*	22.75±4.20	0.005
**Ejection Fraction (%)**	21.34±7.14	46.03±7.32	0.004

Supplementary Table 4. Echocardiography assessment of cardiac structure and function of 22 weeks old *Il13ra1*-deficient and wild-type male mice.

	Il13ra1$^{-/-}$ **(n=7)**	**Wild-type (n=9)**	**P-value**
**Anterior wall diastole (mm)**	0.74±0.10	0.74±0.08	0.938
**Anterior wall systole (mm)**	0.92±0.14	0.98±0.14	0.369
**Posterior wall diastole (mm)**	0.71±0.05	0.82±0.09	0.01
**Posterior wall systole (mm)**	0.84±0.09	1.03±0.10	0.002
**LV diastolic diameter (mm)**	4.22±0.18	3.92±0.28	0.03
**LV systolic diameter (mm)**	3.63±0.29	3.07±0.27	0.002
**LV mass (gr)**	115.37±14.19	109.49±20.74	0.533
**LV volume diastole (mm^3)**	81.48±7.02	70.25±14.63	0.084
**LV volume systole (mm^3)**	57.57±11.28	38.40±7.64	0.001
**Fractional shortening**	13.93±5.03	22.19±4.76	0.005
**Ejection Fraction (%)**	29.70±9.89	45.02±8.01	0.004

<u>Supplementary Table 5.</u> Echocardiography assessment of cardiac structure and function of *Il13ra1*-deficient and wild-type female mice after transverse aortic constriction.

	**Before surgery**		**1 week after surgery**		**3 weeks after surgery**	
	**WT (n=13)**	***Il13ra1*$^{-/-}$ (n=10)**	**WT (n=12)**	***Il13ra1*$^{-/-}$ (n=8)**	**WT (n=12)**	***Il13ra1*$^{-/-}$ (n=8)**
**Anterior wall diastole (mm)**	0.75±0.09	0.71±0.1	0.88±0.13	0.76±0.12	0.93±0.17	0.87±0.11
**P-val**	0.285		0.059		0.448	
**Anterior wall systole (mm)**	1.03±0.14	1.04±0.19	1.20±0.21	1.07±0.14	1.25±0.20	1.14±0.16
**P-val**	0.878		0.146		0.206	
**Posterior wall diastole (mm)**	0.78±0.11	0.79±0.09	3.84±0.24	0.82±0.11	0.96±0.18	0.79±0.09
**P-val**	0.830		0.468		**0.031**	
**Posterior wall systole (mm)**	1.09±0.16	1.13±0.13	2.88±0.27	1.13±0.15	1.22±0.21	1.06±0.124
**P-val**	0.518		0.561		0.080	
**LV diastolic diameter (mm)**	3.81±0.21	3.87±0.36	0.87±0.18	3.79±0.31	3.95±0.30	3.87±0.18
**P-val**	0.351		0.702		0.498	
**LV systolic diameter (mm)**	2.78±0.27	2.79±0.59	1.18±0.21	2.83±0.31	3.02±0.41	2.99±0.40
**P-val**	0.808		0.725		0.853	
**LV mass (gr)**	102.28±15.9	103.32±18.53	125.16±25.46	109.56±15.31	149.34±48.83	127.73±24.76
**P-val**	0.663		0.139		0.265	
**LV volume diastole (mm^3)**	62.41±8.66	64.93±13.83	64.06±9.40	62.90±12.20	68.60±12.06	67.18±6.26
**P-val**	0.327		0.813		0.763	

**LV volume systole (mm^3)**	28.94±7.14	31.58±14.89	32.01±6.91	31.05±8.026	36.69±11.96	37.54±11.79
**P-val**	0.462		0.779		0.876	
**Fractional shortening**	27.53±4.86	28.12±10.0	25.24±4.33	25.46±6.38	23.68±4.94	22.21±6.99
**P-val**	0.786		0.926		0.589	
**Ejection Fraction (%)**	53.90±7.68	53.80±14.88	50.27±7.17	50.4±10.45	47.56±8.74	44.79±12.87
**P-val**	0.961		0.976		0.572	

<u>Supplementary Table 6.</u> Glycolysis cycle reactions change in *Il13ra1*$^{-/-}$ hearts compared to WT controls, according to Integrative Metabolic Analysis Tool (iMAT).

**Reaction name**	**Reaction formula**	**Change**
'glucose-6-phosphate isomerase'	' g6p[c] <=> f6p[c]'	↓
'glyceraldehyde-3-phosphate dehydrogenase'	' g3p[c] + nad[c] + pi[c] <=> 13dpg[c] + h[c] + nadh[c]'	↓
'L-lactate dehydrogenase'	' lac_DASH_L[c] + nad[c] <=> h[c] + nadh[c] + pyr[c]'	↓
'enolase'	' 2pg[c] <=> h2o[c] + pep[c]'	↓
'hexokinase (D-glucose:ATP)'	' atp[c] + glc_DASH_D[c] => adp[c] + g6p[c] + h[c]'	↓
'pyruvate kinase'	' adp[c] + h[c] + pep[c] => atp[c] + pyr[c]'	↓
'catalase A, peroxisomal (ethanol)'	' etoh[x] + h2o2[x] => acald[x] + 2h2o[x]'	-
'phosphoglucomutase'	' g1p[c] <=> g6p[c]'	-
'acetyl-CoA synthetase'	' ac[m] + atp[m] + coa[m] => accoa[m] + amp[m] + ppi[m]'	-
'acylphosphatase'	' 13dpg[c] + h2o[c] => 3pg[c] + h[c] + pi[c]'	-
'Diphosphoglycerate phosphatase'	' 23dpg[c] + h2o[c] => 3pg[c] + pi[c]'	-
'alcohol dehydrogenase (ethanol, NADP), forward reaction'	' etoh[c] + nadp[c] => acald[c] + h[c] + nadph[c]'	-
'fructose-bisphosphatase'	' fdp[c] + h2o[c] => f6p[c] + pi[c]'	-
'aldehyde dehydrogenase (acetylaldehyde, NAD), mitochondrial'	' acald[m] + h2o[m] + nad[m] => ac[m] + 2h[m] + nadh[m]'	-
'Carbamoyl phosphate phosphotransferase, endoplasmic reticulum'	' cbp[r] + glc_DASH_D[r] + h[r] => co2[r] + g6p[r] + nh4[r]'	-
'ethanol monooxygenase'	' etoh[c] + h[c] + nadph[c] + o2[c] => acald[c] + 2h2o[c] + nadp[c]'	-
'alcohol dehydrogenase, forward rxn (ethanol -> acetaldehyde)'	' etoh[c] + nad[c] => acald[c] + h[c] + nadh[c]'	-
'glycerol-3-phosphate dehydrogenase (FAD), mitochondrial'	' fad[m] + glyc3p[c] => dhap[c] + fadh2[m]'	-
'aldehyde dehydrogenase (acetaldehyde, NAD)'	' acald[c] + h2o[c] + nad[c] => ac[c] + 2h[c] + nadh[c]'	-
'Phosphoenolpyruvate carboxykinase (GTP)'	' gtp[c] + oaa[c] => co2[c] + gdp[c] + pep[c]'	-
'Phosphoenolpyruvate carboxykinase (GTP)'	' gtp[m] + oaa[m] => co2[m] + gdp[m] + pep[m]'	-

'aldehyde dehydrogenase (acetaldehyde, NADP)'	' acald[c] + h2o[c] + nadp[c] => ac[c] + 2h[c] + nadph[c]'	-
'glucose-6-phosphate phosphatase, edoplasmic reticular'	' g6p[r] + h2o[r] => glc_DASH_D[r] + pi[r]'	-
'acetyl-CoA synthetase'	' ac[c] + atp[c] + coa[c] => accoa[c] + amp[c] + ppi[c]'	-
'Diphosphoglyceromutase'	' 13dpg[c] <=> 23dpg[c] + h[c]'	-
'phosphofructokinase'	' atp[c] + f6p[c] => adp[c] + fdp[c] + h[c]'	↑
'triose-phosphate isomerase'	' dhap[c] <=> g3p[c]'	↑
'phosphoglycerate mutase'	2pg[c] <=> 3pg[c]'	↑
'phosphoglycerate kinase'	' 3pg[c] + atp[c] <=> 13dpg[c] + adp[c]'	↑
'pyruvate dehydrogenase'	' coa[m] + nad[m] + pyr[m] => accoa[m] + co2[m] + nadh[m]'	↑
'fructose-bisphosphate aldolase'	' fdp[c] <=> dhap[c] + g3p[c]'	↑

Increased flux: "↑", decreased flux: "↓"

<u>Supplementary Table 7.</u> Tricarboxylic acid cycle reactions change in *Il13ra1*$^{-/-}$ hearts compared to WT controls, according to Integrative Metabolic Analysis Tool (iMAT).

**Reaction name**	**Reaction formula**	**Change**
'citrate synthase'	' accoa[m] + h2o[m] + oaa[m] => cit[m] + coa[m] + h[m]'	↓
' 2-oxoglutarate dehydrogenase'	' akg[m] + coa[m] + nad[m] => co2[m] + nadh[m] + succoa[m]'	↓
'ATP-Citrate lyase'	' atp[c] + cit[c] + coa[c] => accoa[c] + adp[c] + oaa[c] + pi[c]'	↓
'Succinate--CoA ligase (GDP-forming)'	' coa[m] + gtp[m] + succ[m] <=> gdp[m] + pi[m] + succoa[m]'	↓
'succinate dehydrogenase'	' fad[m] + succ[m] <=> fadh2[m] + fum[m]'	↓
'malate dehydrogenase, mitochondrial'	' mal_DASH_L[m] + nad[m] <=> h[m] + nadh[m] + oaa[m]'	↓
'fumarase, mitochondrial'	' fum[m] + h2o[m] <=> mal_DASH_L[m]'	↓
'fumarase'	' fum[c] + h2o[c] <=> mal_DASH_L[c]'	↓
'aconitase'	' cit[c] <=> icit[c]'	-
'Isocitrate dehydrogenase (NADP+)'	' icit[m] + nadp[m] <=> akg[m] + co2[m] + nadph[m]'	-
'Isocitrate dehydrogenase (NAD+)'	' icit[m] + nad[m] => akg[m] + co2[m] + nadh[m]'	-
'Citrate lyase'	' cit[c] => ac[c] + oaa[c]'	-
'Isocitrate dehydrogenase (NADP+)'	' icit[x] + nadp[x] => akg[x] + co2[x] + nadph[x]'	-
'isocitrate dehydrogenase (NADP)'	' icit[c] + nadp[c] => akg[c] + co2[c] + nadph[c]'	-
'Aconitate hydratase'	' cit[m] <=> icit[m]'	-
'malate dehydrogenase'	' mal_DASH_L[c] + nad[c] <=> h[c] + nadh[c] + oaa[c]'	↑
'Succinate--CoA ligase (ADP-forming)'	' atp[m] + coa[m] + succ[m] <=> adp[m] + pi[m] + succoa[m]'	↑

Increased flux: "↑", decreased flux: "↓"

Supplementary Table 8. Pyruvate reactions change in *Il13ra1*$^{-/-}$ hearts compared to WT controls, according to Integrative Metabolic Analysis Tool (iMAT).

Reaction name	Reaction formula	Change
' methylglyoxal synthase 2 (from g3p)'	' g3p[c] => mthgxl[c] + pi[c]'	↑
'Propane-1,2-diol:NAD+ 1-oxidoreductase'	' h[c] + lald_DASH_D[c] + nadh[c] => 12ppd_DASH_R[c] + nad[c]'	↑
'D-lactaldehyde dehydrogenase'	' lald_DASH_D[c] + nadp[c] <=> h[c] + mthgxl[c] + nadph[c]'	↑
'alcohol dehydrogenase (L-1,2-propanediol)'	' 12ppd_DASH_S[c] + nad[c] => h[c] + lald_DASH_L[c] + nadh[c]'	↑
'L-lactate dehydrogenase'	' lac_DASH_L[m] + nad[m] <=> h[m] + nadh[m] + pyr[m]'	↑
'alcohol dehydrogenase (L-lactaldehyde)'	' lald_DASH_L[c] + nad[c] => h[c] + mthgxl[c] + nadh[c]'	↑
'aldose reductase (acetol)'	' acetol[c] + h[c] + nadph[c] => 12ppd_DASH_S[c] + nadp[c]'	↑
'lactoylglutathione lyase'	' gthrd[c] + mthgxl[c] => lgt_DASH_S[c]'	↑
'alcohol dehydrogenase (D-1,2-propanediol)'	' 12ppd_DASH_R[c] + nad[c] => h[c] + lald_DASH_D[c] + nadh[c]'	↑
'aldose reductase (methylglyoxal)'	' h[c] + mthgxl[c] + nadph[c] => acetol[c] + nadp[c]'	↑
'lactaldehyde dehydrogenase, mitochondrial'	' h2o[m] + lald_DASH_L[m] + nad[m] => 2h[m] + lac_DASH_L[m] + nadh[m]'	-
'pyruvate carboxylase'	' atp[m] + hco3[m] + pyr[m] => adp[m] + h[m] + oaa[m] + pi[m]'	-
'malic enzyme (NADP), mitochondrial'	' mal_DASH_L[m] + nadp[m] => co2[m] + nadph[m] + pyr[m]'	-
'acetol monooxygenase'	' acetol[c] + h[c] + nadph[c] + o2[c] => 2h2o[c] + mthgxl[c] + nadp[c]'	-
'alcohol dehydrogenase (D-lactaldehyde)'	' lald_DASH_D[c] + nad[c] => h[c] + mthgxl[c] + nadh[c]'	-
'acetyl-CoA hydrolase'	' accoa[c] + h2o[c] => ac[c] + coa[c] + h[c]'	-
'D-lactate dehydrogenase'	' lac_DASH_D[c] + nad[c] <=> h[c] + nadh[c] + pyr[c]'	-
'lactaldehyde dehydrogenase, mitochondrial'	' h2o[m] + lald_DASH_D[m] + nad[m] => 2h[m] + lac_DASH_D[m] + nadh[m]'	-
'lactaldehyde dehydrogenase'	' h2o[c] + lald_DASH_L[c] + nad[c] => 2h[c] + lac_DASH_L[c] + nadh[c]'	-
'malic enzyme (NADP)'	' mal_DASH_L[c] + nadp[c] => co2[c] + nadph[c] + pyr[c]'	-

'lactaldehyde dehydrogenase'	' h2o[c] + lald_DASH_D[c] + nad[c] => 2h[c] + lac_DASH_D[c] + nadh[c]'	-
'malic enzyme (NAD), mitochondrial'	' mal_DASH_L[m] + nad[m] => co2[m] + nadh[m] + pyr[m]'	-
'hydroxyacylglutathione hydrolase, mitochondrial'	' h2o[m] + lgt_DASH_S[m] => gthrd[m] + h[m] + lac_DASH_D[m]'	-
'L-Lactate dehydrogenase, cytosolic/mitochondrial'	' 2ficytC[m] + lac_DASH_L[c] => 2focytC[m] + 2h[c] + pyr[c]'	-
'hydroxyacylglutathione hydrolase'	' h2o[c] + lgt_DASH_S[c] => gthrd[c] + h[c] + lac_DASH_D[c]'	-
'acetone monooxygenase'	' acetone[c] + h[c] + nadph[c] + o2[c] => acetol[c] + h2o[c] + nadp[c]'	-
'Propane-1,2-diol:NADP+ 1-oxidoreductase'	' h[c] + lald_DASH_D[c] + nadph[c] => 12ppd_DASH_R[c] + nadp[c]'	↓
'D-Lactaldehyde:NADP+ 1-oxidoreductase'	' h[c] + mthgxl[c] + nadph[c] => lald_DASH_D[c] + nadp[c]'	↓
'D-Lactaldehyde:NAD+ oxidoreductase (glutathione-formylating)'	' gthrd[c] + lald_DASH_D[c] + nad[c] <=> h[c] + lgt_DASH_S[c] + nadh[c]'	↓
'D-Lactaldehyde:NAD+ 1-oxidoreductase'	' h[c] + mthgxl[c] + nadh[c] => lald_DASH_D[c] + nad[c]'	↓
'methylglyoxal synthase'	' dhap[c] => mthgxl[c] + pi[c]'	↓

Increased flux: "↑", decreased flux: "↓"
